# The Effect of Pivotal Response Treatment in Children with Autism Spectrum Disorders: A Non-randomized Study with a Blinded Outcome Measure

**DOI:** 10.1007/s10803-016-2916-0

**Published:** 2016-11-04

**Authors:** E. A. Duifhuis, J. C. den Boer, A. Doornbos, J. K. Buitelaar, I. J. Oosterling, H. Klip

**Affiliations:** 1 Karakter Child and Adolescent Psychiatry, Postbus 68, 6710 BB Ede, The Netherlands; 2 Karakter Child and Adolescent Psychiatry, Vriezenveenseweg 213, 6700 AP Almelo, The Netherlands; 3 Karakter Child and Adolescent Psychiatry University Center, Reinier Postlaan 12, 6525 GC Nijmegen, The Netherlands; 4 Department of Cognitive Neuroscience, Donders Institute for Brain, Cognition and Behaviour, Radboudumc, P.O. Box 9101 (204), 6500 HB Nijmegen, The Netherlands

**Keywords:** Autism, Pivotal response treatment, Children, ADOS

## Abstract

Purpose of this quasi-experimental trial was to investigate the effect of Pivotal response treatment (PRT) versus treatment as usual (TAU) on autism symptoms. Children with autism spectrum disorder (ASD), aged 3–8 years, received either PRT (n = 11) or TAU (n = 13). Primary outcome measure was the total score on the Autism Diagnostic Observation Schedule at pre- and posttreatment. Additionally, general problem behavior and parental stress levels were measured. Children in the PRT condition improved on the primary outcome measure compared to the TAU group with a small effect size [partial η^2^ = 0.22 (95 % CI 0.00–0.46)]. Neither group demonstrated significant changes in the secondary outcomes. This study suggests that PRT may improve autism symptoms in children with ASD over TAU.

## Introduction

Autism spectrum disorders (ASD) are severe neurodevelopmental disorders, characterized by impairments in social interaction, verbal and non-verbal communication, along with a restricted repertoire of activities and interests (American Psychiatric Association 2013). A systematic review reported a prevalence rate of 17 per 10,000 people for the core syndrome (autistic disorder) and a rate of 62 per 10,000 people for all ASDs (Elsabbagh et al. 2012). The number of children diagnosed with ASD has risen dramatically in recent years, forcing services to expand their treatment programs. Outcomes for children with ASD appear to have substantially improved with the provision of early intensive behavioral interventions (EIBIs) (Rogers and Vismara 2008; McConachie and Diggle 2007; Howlin et al. 2009; Reichow 2011; Warren et al. 2011).

One intervention showing promising results is the Lovaas Model of Applied Behavior Analysis. This model employs Discrete Trial Training (DTT) or EIBI techniques that involve breaking down behaviors into their smallest functional units and presenting them in series. It is a highly structured, intensive behavioral treatment, delivered by therapists, that requires 35–40 h per week, for two or more years (Lovaas 1987; Reichow 2011).

Another potentially effective intervention based on a behavioral approach is known as pivotal response treatment (PRT) (Simpson 2005; Masiello 2003; National Autism Center 2009). The goal of PRT is to teach children (or adults) to respond to the many learning opportunities and social interactions that occur in their natural environment and to increase their motivation to communicate (Koegel et al. 1989, 1999a; Koegel and Koegel 2006). PRT focuses on core pivotal areas (for example, motivation, self-initiation, multiple cues and self-management), improvements of which are thought to result in widespread gains in untargeted areas, such as reduction of behavioral problems (Koegel et al. 2005). PRT is characterized by a naturalistic behavioral approach, where interventions are primarily embedded in functional activities that are less structured as compared to DTT, and more child-focused, using intrinsically-related rewards. Techniques include following the child’s lead, using clear instructions (prompts), providing immediate effective contingent rewards, making use of direct and natural reinforcers (rewards), reinforce good attempts and interspersing tasks.

PRT has a number of advantages compared to DTT and EIBI. These benefits include using natural reinforces which improve generalization. Conversely, the artificial or unrelated reinforces used in DTT can prevent generalization, lead to cue dependency and rote responding (Vismara and Rogers 2010). Furthermore PRT is expected to increase parental self-efficacy because of their explicit and pronounced involvement (Koegel et al. 2001), in contrast to DTT that is delivered by therapists. PRT is carried out in a child’s everyday environment, whereas the adult-directed nature of the instruction and strict stimulus control in DTT may limit the spontaneous application of skills. In PRT, the explicit involvement of parents and other individuals in the child’s daily life decreases the amount of services needed, resulting in less cost intensive treatments. Parents are trained in using PRT techniques as much as possible in everyday life. In contrast, DTT treatment is delivered by experienced therapists, with rigorous levels of training and supervision. Finally, DTT has been criticized for its punitive procedures (Vismara and Rogers 2010).

PRT purports to be an evidence-based treatment (Rogers and Vismara 2008) and the National Autism Center recognizes it as an established intervention (2009). Indeed, several studies show positive results. For example, Hardan et al. (2015) conducted an RCT to compare PRT in a group of parents of children aged 2–6 years (n = 27) with a parent psychoeducation group (n = 26). Improvements were observed in frequency of utterances and adaptive communication skills, but not in autism symptoms on the Social Responsiveness Scale (SRS). A 3-month follow-up study of the parent PRT group without the psychoeducation group showed retainment of these gains, although it was unclear whether the parents provided PRT in this period (Gengoux et al. 2015). Another study provided a community-based early intervention study based on PRT (Smith et al. 2015). A group of 118 children (mean age = 49 months, SD = 9.4) was divided into three groups, being very low IQ <40, moderately low IQ 40–69 and higher IQ >70 and followed a 1-year PRT program. No control group was used. Communication and adaptive behavior improved for all groups. Behavior problems (CBCL) only decreased in the high IQ group. There was no effect on parental stress. Mohammadzaheri et al. (2014), conducted an RCT comparing PRT and a structured ABA approach in a school setting (N = 30, 6–11 years) and found that PRT was significantly more effective in improving targeted areas (mean length of utterance) and untargeted areas (pragmatic skills) after 3 months of intervention.

Notwithstanding the positive results of PRT with respect to increased self-initiation, collateral improvements in language and communication skills and improved affect and play skills for the majority of children with ASD, as well as reduced maladaptive behavior with some children with ASD (Koegel and Koegel 2006; Randolph et al. 2011; Robinson 2011), most studies have methodological shortcomings. Some studies include few participants (N = 3, N = 2) (Randolph et al. (2011); Voos et al. (2012); Steiner et al. (2013). Pierce and Schreibman (1995), had no control condition (Baker-Ericzén et al. (2007); Minjarez et al. (2011)), or did not incorporate all PRT techniques (Verschuur et al. 2014). Cardogan and McCrimmon (2015) did a systematic review of the quality of the studies into PRT and recommended the researchers to compare different interventions, use longitudinal designs, describe methodologies more thoroughly and implement greater adherence to treatment fidelity and so enhance the quality of the studies and strengthen the conclusions.

The aim of the present study was to investigate the effectiveness of PRT treatment compared to TAU in The Netherlands with a relatively large group of children with ASD and including a control group after 6 months of intervention, using the Autism Diagnostic Observation Schedule (ADOS) as the main outcome measure rated by blinded raters. Secondary aims were to evaluate whether PRT, compared to TAU, would be associated with greater reduction of parental stress levels and general problem behaviors of the children. A combination of parent reported instruments and therapist reported instruments were utilized.

## Methods

### Study Design

This was a multicenter, non-randomized trial using repeated measures to evaluate PRT versus TAU in young children (aged 3–8 years), with ASD in The Netherlands. This study was approved by the Institutional Review Board of the treatment institute. Randomization at individual or site level was not possible for pragmatic reasons, being availability of PRT therapists at only one location and research budget constraints. Parents completed questionnaires at baseline (T1), 3 months (T2), and 6 months (T3). Baseline measures (T1) were administered before PRT or TAU commenced. The primary outcome (severity of autism symptoms measured on the ADOS) was measured at baseline (T1) and after 6 months (endpoint; T3), whereas the secondary measures were collected three times; at baseline, after 3 months and at 6 months (endpoint; T3).

### Recruitment of Participants

Patients eligible for participation in the study were recruited between May 2012 and January 2013, from the outpatient clinics of six sites of a large Child and Adolescent Psychiatry Center (Karakter), in the Eastern part of The Netherlands. Eligibility criteria for the study were as follows: (1) 3–8 years old; (2) DSM-IV classification of ASD, as confirmed by a child psychiatrist, based on a multidisciplinary assessment that included a psychiatric observation, developmental assessment, parental interview, and daycare or school questionnaires; (3) Nonverbal Intelligence Quotient (NVIQ) score above 50, measured using either the Wechsler Intelligence Scale for Children III—NL (WISC-III) (Wechsler 1991), the Wechsler Primary Scale of Intelligence—R (WPPSI-R) (Wechsler 1989), the Snijders-Oomen Nonverbal Intelligence Test—R (SON-R) 2½–7 (Tellegen 1996), or the Mullen Scales of Early Learning (Mullen 1995); (4) Ability to speak one word as a minimal level of language proficiency; (5) Dutch speaking parent(s); (6) Parents motivated to participate in the study and willing to sign informed consent. Exclusion criteria included non-regulated, comorbid attention deficit hyperactivity disorder (ADHD) and receiving other specific individual therapies focusing on improving social-communication, for example speech and language therapy.

Children were referred from primary, secondary, and tertiary health care services. Children included in the PRT group were recruited in Ede and Nijmegen. Children in the TAU group were recruited from Ede, Nijmegen, Zwolle, Apeldoorn, Hengelo and Almelo. All locations belong to the same child and adolescent psychiatric hospital and use the same clinical protocols. The largest distance between two places is less than 65 miles. Patients in the TAU group did not have contact with PRT therapists. Unlike the United States, for example, where treatment intensity may range from 10 to 50 h a week, the health care system in The Netherlands is organized and financed following the principle: “as short as possible and as intensive as needed”.

### Interventions

In the PRT group participants and their parents received PRT training according to a written protocol that included 20 sessions of 45 min duration each over a period of 6 months. PRT was conducted by certified PRT therapists who were extensively trained to reach a fidelity score of over 80 %. In the first three sessions the therapist demonstrated the PRT techniques to the parent(s), while the child became more familiar with the therapist. In addition, written information about the therapy was provided and discussed with parents. Individual goals were dependent on the level of development of each individual patient. One child, for example, learned how to speak using an increased range of vocabulary in sentences, while another child learned how to ask meaningful questions and to protest appropriately. PRT techniques are similar for all age groups. On the fourth, ninth, fourteenth and nineteenth sessions, the therapist viewed the previous taped sessions in conjunction with the parent(s) in order to provide more intensive feedback. Parents learned to set goals and also how to practice these skills at home. In the fifth and subsequent sessions, parents played with their child using the PRT techniques, while receiving feedback and real live coaching from the therapist. Possible ways for applying PRT techniques in various everyday situations at home were discussed and parents were instructed to apply these techniques as many times as possible, thus enormously increasing exposure. Videotapes of all parents were scored and parent treatment fidelity was measured.

TAU consisted of parent psycho education and parent mediation therapy ranging from low-frequency sessions with a psychologist (for example, 1 h per month) to intensive parental training set up in their home environment (for example, twice a week, of 75 min duration, over 20 weeks) by a family worker. Some parents received psycho education in a group setting with other parents (generally once a week, over a 5-week period) and, in some instances, followed by individual parental guidance.

### Measures

#### Primary Outcome Measure

##### Autism Diagnostic Observation Schedule (ADOS)

The primary outcome measure was the total score on the ADOS. The ADOS is a semi-structured, standardized, observational assessment designed for use with individuals referred with a differential diagnosis of ASD. It provides an indication of social-communicative (dis)abilities, as observed by a trained professional (Gotham et al. 2008). The instrument showed good inter-rater reliability, internal consistency and test–retest reliability on item level, domain level and diagnostic level for autism and non-spectrum disabilities (Lord et al. 2000). In the current study the ADOS was administered and coded by psychologists trained up to research reliability. The ADOS consists of four modules, each requiring 35–60 min to administer. The individual being evaluated completes one single module depending on his or her expressive language level and chronological age. Module 1 is intended for children who do not consistently use phrase speech (10 activities, 29 scores). Module 2 is administered to children who use phrase speech but are not verbally fluent (14 activities, 28 scores). Module 3 is used with fluently speaking children and young adolescents (14 activities, 28 scores). Scores are calculated based on observed behavior. Most item scores fall within the range of 0 (normal behavior) to 2 or 3 (abnormal behavior) (Lord et al. 2000). In this study, children were administered either Module 1, 2, or 3, in accordance with their age and level of spoken language at baseline (T1) and again at the end of the study (T3; 6 months). Using the revised algorithms (Gotham et al. 2007), the ADOS domain totals for Social Affect (SA) and Restricted and Repetitive Behavior (RRB) factors were generated. The SA domain includes items which give an indication of social communicative (dis)abilities, whereas the RBB domain observes restrictive and repetitive behavior. In addition, the sum of these two scales was calculated as the total score (SARRB) and this was used as a primary outcome measure.

Gotham et al. (2009) developed calibrated severity scores (CSS) to measure the relative severity of autism-specific features. They found that calibrated severity scores had more uniform distributions across developmental groups and were less influenced by participant demographics than raw totals. They concluded that this metric should be useful in comparing assessments across modules and time. In addition to the SARBB, calibrated severity scores were calculated to make meaningful comparisons across participants and pre-post within-subject comparisons (Gotham et al. 2009). Hus et al. (2014) proposed to use separate calibrations of each domain (SA and RRB) in order to be able to provide a clearer picture of ASD dimensions. We also calculated the SA-CSS and the RRB-CSS (Hus et al. 2014).

#### Secondary Outcome Measures

##### Social Responsiveness Scale (SRS)

Complementary to observed autism symptoms by a professional, we applied the SRS (SRS; Dutch version; Roeyers and Thys 2010). This is a questionnaire to be completed by parents/caregivers, and it is developed to measure the severity of social and communicative symptoms in everyday social situations in children and adolescents without intellectual disabilities, 4–17 years old (Constantino and Gruber 2005). All 65 items are scored on a four-point scale (0 = never, 4 = almost always true). A higher score is indicative of greater problems. The SRS has five subscales: Social Awareness (ability to detect social cues); Social Cognition (ability to interpret social cues once they are identified); Social Communication (includes expressive social communication); Social Motivation (the extent to which a respondent is generally motivated to engage in social-interpersonal behavior); Autistic Mannerisms (includes stereotypical behaviors or highly restricted interests, characteristics associated with autism). The raw total score and the original scores on the individual scales were converted to a standard score (T-score). The Cronbach’s alpha of the SRS was assessed from three different samples (Dutch sample: N = 1,324, Flemish sample: N = 370, autism spectrum sample: N = 238) and ranged from 0.92 to 0.95. Other psychometric criteria were reported to be sufficient to good (Roeyers et al. 2011).

##### Child Behavior Checklist (CBCL)

Behavioral problems were measured using parents’ ratings on the Dutch translation of the Child Behavior Checklist (CBCL). This is a widely used standardized questionnaire for those aged either 1½−5 or 6–18 years (Achenbach and Rescorla 2000). The CBCL is a parent rating scale which measures children’s general problem behavior and internalizing and externalizing behavior, while more specific problem behaviors are assessed with supplementary scales. In the analyses of this study, three major scales in the CBCL were focused on: The total scale, externalizing behavior, and internalizing behavior. Cut-off scores for clinically elevated symptoms are based on T-scores ≥68 (Achenbach and Rescorla 2000). Internal consistencies (Cronbach’s Alpha) for the Dutch version were found to be >0.90 (Verhulst et al. 1996).

##### Nijmegen Parental Stress Index—Short Version (NOSI-K)

Parental stress was measured with the Nijmegen Parental Stress Index—short version (NOSI-K). The NOSI-K is a short version of the NOSI (full parental stress index) that can be completed by the parent in approximately 5 min. It consists of 25 items that reflect ten scales, namely: Competence, attachment, depression, health, adjustment, mood, distractibility, fussiness, positive ratification and acceptance (De Brock et al. 1992). Cronbach’s alpha of the NOSI-K is between 0.92 and 0.95. Construct validity of the NOSI-K has not been investigated (NJI, 2015).

##### Cognitive Ability

Non-verbal IQ’s were based on subscales of the WISC-III-NL, the WPPSI-III-NL, the Mullen Scales of Early Learning or on the SON-R 2½–7, a Dutch widely used non-verbal intelligence test. NVIQ’s based on the Mullen were calculated as follows: age-equivalent scores from the Fine Motor and Visual Reception domains were averaged, then divided by the child’s chronological age, and multiplied by 100 (Bishop et al. 2011).

##### Background Information

Baseline demographic data were gathered using the Intake Questionnaire developed by Karakter Child and Adolescent Psychiatry Department. It is a 108-item questionnaire which elicits demographic information, including age and gender of the parent and child, socioeconomic status (SES), level of education of parents and siblings, psychological and psychiatric illnesses in the family, details about the pregnancy, birth, development, health, healthcare, school, sleep, etc. All patients and their parents (0–18 years) have to complete this questionnaire prior to their first appointment at Karakter. The items selected for analysis are listed in Table 1 below. A high level of education means academic and higher vocational education, middle level means intermediate vocational education and a low level means lower vocational education.

### Blinding

Psychologists administering the ADOS (primary outcome measure) were blind to the treatment condition. Other measures were rated by computer. Parents were not blind to treatment condition.

### Sample Size

To detect a difference of 3.0 (±3.0) on the ADOS score after 6 months between the PRT group and TAU group, with a two-sided five percent significance level and a power of 80 percent, a sample size of 16 patients per group was necessary, given an anticipated dropout rate of 10 percent. To recruit this number of patients, an inclusion period of 6 months was anticipated. Because of difficulties with the recruitment of patients, the inclusion was extended by 6 months and then terminated for budgetary reasons. As a result, a total of 24 patients were included in the study.

### Statistical Analyses

In order to assess possible differences in the demographic characteristics between the TAU and PRT groups, Chi square test was conducted for categorical data. For each group, the frequencies and percentages were reported. For continuous data, mean ± standard deviation were reported. An independent-samples *t* test was run to determine differences in the demographic variables between PRT and TAU. Outliers were assessed by inspection of a boxplot, normality was tested using the Shapiro–Wilk test (p > .05), and homogeneity of variances was determined by the Levene’s test. All patient scores were analyzed using the intention to treat analysis model.

To determine the effect of PRT over time, a two-way repeated measures analysis of variance (ANOVA) was run with group as between subjects factor (PRT versus TAU) and time as within-subjects factor on the ADOS total score [SARRB, the two subscales (SA and RRB)], the calibrated scores as well as the SRS, CBCL and NOSI-K. A significant group by time interaction effect implicates treatment effect. Analysis of the studentized residuals showed that they were normally distributed, as assessed by the Shapiro–Wilk test of normality, and there were no outliers, as assessed by no studentized residuals being greater than ±3 standard deviations. The sphericity of the interaction term was checked using Mauchly’s test of sphericity (p > .05). For these analyses, Statistical Package for the Social Sciences (SPSS) for Windows, Version 22.0 (SPSS Inc., Chicago, Ill, USA) software was used. An interim analysis was not scheduled.

## Results

### Participant Flow and Baseline Characteristics

**Fig. 1:**
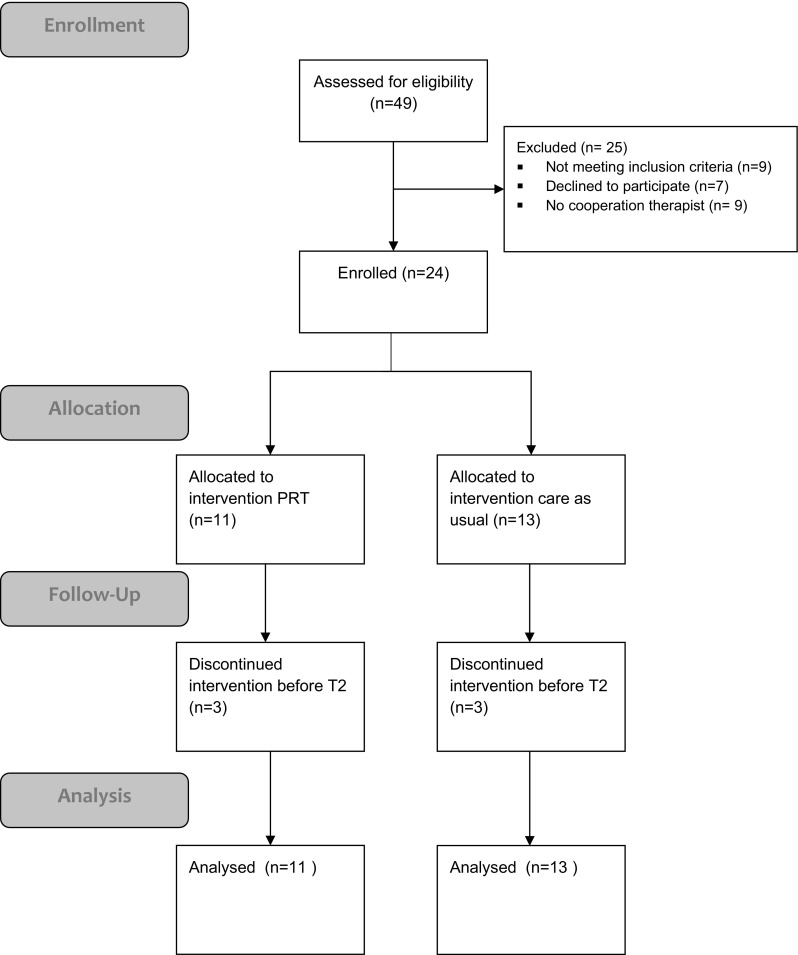
The flow of participants through the study

A total of 49 patients were screened for eligibility and, of those, 24 were enrolled in this study (Fig. 1). Nine patients did not meet the inclusion criteria, seven declined to participate, while a further nine patients failed enrollment due to commencing therapy prior to completion of baseline measurements. 11 patients received PRT treatment and 13 patients received TAU. In the PRT group, three patients withdrew from therapy prior to the midpoint measurements (following 3 months of therapy). In the TAU group, another three patients discontinued treatment before the midpoint measurements. All patient scores were analyzed (using the intention to treat analysis model), as all questionnaires were completed.

Table 1 describes the target behavior of the children in the PRT group. Most children were taught to be more socially directed and to ask appropriate questions as social initiatives. Whereas most children could speak in sentences of any kind at pre-treatment, one child spoke in separate words and the target behavior was directed at communication in sentences. Two children were taught self-management skills.

**Table 1:** PRT Target behavior in relation to the age of the children

Target behavior	Child	Total number of children	Age (months)	Age (months) Mean ± SD
Social directedness^a^	13^c^, 20, 23, 27, 34, 16, 19, 31, 33	9	49, 50, 51, 66, 57, 68, 82, 86, 61	63.3 ± 20.3
One word	23	1	51	
Two word utterance	23	1	51	
Asking for an object	11, 13, 20, 23, 27, 34, 16, 32, 19, 31, 33	11	100, 49, 50, 51, 66, 57, 68, 89, 82, 86, 61	69.0 ± 22.7
Asking for help	11, 13, 20, 23, 27, 34, 16, 32, 19, 31, 33	11	100, 49, 50, 51, 66, 57, 68, 89, 82, 86, 61	69.0 ± 22.7
Wh-questions^b^	11, 13, 20. 23, 27, 34, 16, 33	8	100, 49, 50, 51, 66, 57, 68, 61	62.8 ± 22.7
Protest	11, 13, 20, 23, 27, 34, 16, 32, 19, 31, 33	11	100, 49, 50, 51, 66, 57, 68, 89, 82, 86, 61	69.0 ± 22.7
By questions/conversation	11, 20, 27, 34, 16, 32, 19, 31	8	100,50,66,57,68,89,82,86	74.8 ± 25.9
Making comments	11, 20, 16, 32, 19, 31, 33	7	100, 50, 68, 89, 82, 86, 61	76.6 ± 27.6
Multiple cues	11, 13, 20, 23, 27, 34, 16, 32, 19, 31, 33	11	100, 49, 50, 51, 66, 57, 68, 89, 82, 86, 61	69.0 ± 22.7
Self-management	11, 32	2	100, 89	94.5 ± 43.8

^a^Social directedness: pointing, giving, sharing, grabbing the hand, joint attention

^b^Wh-questions: Who, What, Where questions

^c^These are the participant numbers

The TAU group consisted of two kinds of treatment, being psycho-education and/or parent guidance or intensive parental home training (Table 2).

**Table 2:** Treatment sessions TAU and PRT

Treatment	Total number of children	Total number of sessions	Average number of session	Range of treatment hours at institution	Average treatment hours at institution
Parent psycho education, parent guidance	5	8, 7, 5, 15, 3	7.6	2.3–11.3	5.7
Intensive parental home training	8	24,15, 37, 33, 20, 21, 29, 26	25.6	11.3–27.8	19.2
Pivotal response treatment	11	11, 12, 13, 8 times 20	17.8	8.3–15	13.4

As indicated in Table 3, in both groups, the majority of participants were boys, approximately one-third of all children had a low IQ, the educational level of the parents appeared to be somewhat higher in the PRT group, and three children in the control group were taking medication (methylphenidate, atomoxetine or dextroamphetamine in the PRT group and one risperidone in the TAU group). However, none of the differences in demographics between the two groups were significant at pretest. The number of treatment sessions was equal for both groups (mean ± SD PRT 17.8 ± 3.7; TAU 18.7 ± 11.0).

**Table 3:** Patient and demographic characteristics of PRT versus treatment as usual group

	Treatment as usual (n = 13)		PRT (n = 11)		p value^a^
	n	%	n	%	
Sex					
Female	3	23.1 %	1	9.1 %	0.360
Male	10	76.9 %	10	90.9 %	
Age					
3–5 years	7	53.8 %	7	63.6 %	0.628
6–9 years	6	46.2 %	4	36.4 %	
Age start	5.7 ± 2.2		5.9 ± 1.5		0.992
Total IQ					
TIQ 50–85	3	23.1 %	4	36.4 %	0.691
TIQ 85–115	6	46.2 %	5	45.5 %	
TIQ 115–130	4	30.8 %	2	18.2 %	
Education mother^c^					
Low	2	15.4 %	0	0.0 %	0.267
Average	5	38.5 %	3	27.3 %	
High	6	46.2 %	8	72.7 %	
Education father^c^					
Low	2	15.4 %	2	18.2 %	0.178
Average	7	53.8 %	2	18.2 %	
High	4	30.8 %	7	63.6 %	
Co morbidity					
ADHD	2	15.4 %	2	18.2 %	0.558
Cerebr. parese	1	7.7 %	0	0.0 %	
Epilepsy	0	0.0 %	1	9.1 %	
No	10	76.9 %	8	72.7 %	
Unknown	1	7.7 %	4	36.4 %	
Medication					
No	12	92.3 %	7	63.6 %	0.085
Yes	3	23.1 %	1	9.1 %	

	Treatment as usual (n = 13)		PRT (n = 11)		p value^b^
	Mean ± SD		Mean ± SD		
Total IQ	98.4 ± 18.6		94.9 ± 22.3		0.681
Total NVIQ	95.5 ± 17.6		93.0 ± 21.2		0.751

^a^Chi-square

^b^Independent sample *t* test

^c^Education: Low = primary school, Average = high school, High = university and higher professional education

### Primary Outcome Measure: Autism Symptoms

In the TAU group one ADOS score was missing. Before the interventions commenced, children in the PRT group had higher total scores on the ADOS (mean 14.8 ± 5.0) than children in the TAU group (mean 7.3 ± 3.8), which suggests that children in the PRT group had more autistic symptoms (see Table 4). By the end of the treatment, children in the PRT group had improved on these scores (mean 13.2 ± 5.0), whereas autistic symptoms increased in the TAU group (mean 9.0 ± 3.6). This results in a statistically significant interaction between group and time on the total ADOS scores, F(1, 21) = 5.737, p = .026, partial η^2^ = 0.215 (90 % CI 0.015–0.425). As illustrated in Table 4, none of the subscales on the ADOS demonstrated a significant interaction effect between group and time.

**Table 4:** Main effects and interaction effects on the ADOS between pre-treatment and post-treatment for the control group and PRT group

	TAU group (n = 12)		PRT group (n = 11)		Interaction-effect^c^		
	Pre-treatment Mean ± SD	Post-treatment Mean ± SD	Pre-treatment Mean ± SD	Post-treatment Mean ± SD	F	p	η^2^
ADOS							
Subscale SA	6.9 ± 3.6	8.4 ± 3.6	13.2 ± 4.1	11.9 ± 4.8	3.974	.059	.159
Subscale RRB	0.4 ± 1.2	0.6 ± 0.5	1.6 ± 1.7	1.3 ± 1.2	1.052	.317	.048
Total score SARRB	7.3 ± 3.8	9.0 ± 3.6	14.8 ± 5.0	13.2 ± 5.0	5.737	.026	.215
Calibrated severity^a^	3.8 ± 2.1	4.8 ± 2.5	7.2 ± 2.1	6.6 ± 2.1	4.871	.039	.188
Calibrated severity^b^ Subscale SA	5.0 ± 2.4	5.7 ± 2.4	8.2 ± 1.7	7.6 ± 2.2	2.592	.122	.110
Calibrated severity^b^ Subscale RRB	2.0 ± 2.1	2.8 ± 1.5	4.5 ± 3.1	4.2 ± 2.8	1.481	.237	.066

*SA* social affect, *RRB* repetitive restrictive behavior

^a^Calculation based on Gotham et al. (2009)

^b^Calculation based on Hus et al. (2014)

^c^p < 0.05

The calibrated severity scores show the following results: PRT prescore (7.2 ± 2.1) and TAU prescore (3.8 ± 2.1), PRT postscore (6.6 ± 2.1) TAU postscore (4.8 ± 2.5). This results in a statistically significant interaction between group and time on the ADOS calibrated severity scores, F(1, 21) = 4.871, p = .039 partial η^2^ = 0.188 (90 % CI 0.006–0.400). We also calculated the calibrated severity scores for the subscales SA and the RRB and found no interaction effect between group and time.

### Secondary Outcome Measures

In Table 5, the results are presented for the group by time-repeated measures analyses for the total scores on the SRS, CBCL, and NOSI-K. These instruments were measured three times: before treatment, after 3 months and again at 6 months. The analysis did not show any treatment effects (see Table 5). Only for the subscale Communication (SRS), a statistically significant interaction was found between group and time F(2, 36) = 3.931, p = .044, partial η^2^ = 0.179 (90 % CI 0.011–0.329).

**Table 5:** Main effects and interaction effects on the NOSIK, CBCL and SRS between the three time points for the control group and PRT group

	TAU group (n = 11)			PRT group (n = 10)			Interaction-effect^a^		
	Pre-treatment	3 Months treatment	6 Months treatment	Pre-treatment	3 Months treatment	6 Months treatment	F	p	η2
	Mean ± SD	Mean ± SD	Mean ± SD	Mean ± SD	Mean ± SD	Mean ± SD			
NOSIK									
Total score	87.5 ± 27.4	94.4 ± 32.4	88.5 ± 31.7	71.7 ± 20.7	73.5 ± 18.5	75.0 ± 18.8	.625	.486	.032
CBCL									
Internalizing T score	69.4 ± 9.6	68.8 ± 10.8	67.1 ± 12.8	63.9 ± 9.3	64.9 ± 7.3	61.6 ± 6.1	.263	.770	.014
Externalizing T score	67.4 ± 9.9	68.2 ± 11.5	69.2 ± 11.0	62.0 ± 7.1	62.1 ± 7.8	60.2 ± 7.3	1.198	.313	.059
Total T score	69.9 ± 9.2	69.3 ± 11.1	69.0 ± 10.9	65.6 ± 6.5	65.0 ± 7.7	61.6 ± 6.4	1.095	.345	.054
SRS									
Social motivation	14.2 ± 4.9	13.1 ± 6.5	12.7 ± 4.9	14.5 ± 5.3	12.9 ± 5.1	13.8 ± 5.0	.454	.639	.025
Social awareness	11.9 ± 1.6	12.0 ± 2.9	11.0 ± 3.5	13.2 ± 2.9	12.5 ± 3.7	12.8 ± 3.1	.539	.588	.029
Social cognition	18.9 ± 4.3	19.6 ± 4.9	17.9 ± 4.7	17.5 ± 5.5	16.3 ± 4.8	15.6 ± 4.4	.834	.442	.044
Social communication	26.2 ± 4.8	27.0 ± 5.2	25.4 ± 7.6	33.5 ± 8.1	28.2 ± 5.6	27.9 ± 3.9	3.931	.044	.179
Autistic mannerisms	15.0 ± 4.2	14.0 ± 5.4	14.0 ± 5.6	16.2 ± 5.8	14.6 ± 5.2	14.3 ± 4.5	.112	.894	.006
Total score	141.8 ± 10.5	139.1 ± 13.5	139.8 ± 15.4	149.3 ± 18.6	139.3 ± 11.9	138.8 ± 10.4	1.548	.227	.079

^c^p < 0.05

Ten parents reached a treatment fidelity of more than 80 %, only one parent reached a fidelity of 73 %.

## Discussion

This study investigated the effectiveness of PRT in comparison to TAU on autism symptoms and general problem behaviors of children with ASD, as well as parental stress levels. The non-randomized assignment limits the conclusions that can be drawn, but we found a significantly positive treatment effect of PRT on autism symptoms, with slight improvements in the PRT group and deterioration in the TAU group.

One explanation for the differences between the TAU versus PRT outcomes is that PRT prevents the mechanism of growing into deficit: PRT may prevent widening of the gap between normal and autistic development. This can be explained by the focus of the treatment. TAU is more specifically focused on teaching the social environment how to cope with the child with ASD and his/her symptoms, whereas PRT is aimed at decreasing autism symptoms rather than accepting them. PRT challenges the child to improve his communication.

The small improvement in the PRT group might also be due to regression to the mean i.e. the tendency for outcome scores to align with a population mean over time. Our study is one of the few that used the ADOS as an outcome measure for the effect of PRT and has a larger sample size than most PRT studies with a control group (Voos et al. 2012; Steiner et al. 2013). Previous studies investigating interventions for children with ASD using the ADOS as a primary outcome measure found significant improvement (Aldred et al. 2004; Green et al. 2010). However, the intervention was not PRT, but rather a communication-based parent mediation treatment program.

There was no improvement on the autism symptoms rated by parents (SRS). Only the communication subscale in the PRT group showed a significant result. This is consistent with the findings of Gengoux et al. (2015) who found no significant changes on the SRS standard scores in a PRT parent training group of children aged 2–6 years. Smith et al. (2015) used the Nova Scotia early intensive behavior intervention model, incorporating PRT and found a decrease in autism symptoms on the SRS for children (mean age 50 months) with a TIQ > 50 (n = 45). In contrast with the ADOS, the SRS is a parent reported measure based on observations of the parents in daily practice, allowing for more variability and subjectivity, whereas the ADOS is used in a standardized setting by more objective trained professionals.

The theoretical model of PRT purports that targeting pivotal skills using PRT techniques results in widespread improvements in other aspects of functioning. This could not be confirmed in this study (CBCL). This is consistent with some other (Simpson 2005; Masiello 2003) but not all prior findings (Koegel et al. 2001).

No difference in parental stress at the beginning or end of therapy in both treatment groups was identified. This is consistent with other findings (Oono et al. 2013). It is possible that parental stress was affected by other factors, such as their own individual characteristics or life events. Measuring the amount of positive affect or parent–child positive interaction may be a more sensitive indicator of parental stress (Minjarez et al. 2013), and could be considered as a meaningful type of outcome measure in future research.

### Limitations

The results of this study should be interpreted in the context of some limitations. First, randomization was not possible. Although, both groups were comparable in respect of age, intelligence, sex and SES, the pre-treatment scores on the ADOS and SRS were significantly higher at the beginning of the study in the PRT group compared to the TAU group. Indeed this initial difference in autism severity is a problem, so results should be cautiously interpreted. Secondly, the study was limited by its small sample size. Despite this, significant findings were found on the ADOS. The small sample size may be a reason why no gains were evidenced with respect to other measures.

Thirdly, as PRT therapists were only available in one location, allocation bias may have been a problem. However, it’s unlikely that differences in outcome can be attributed to varying geographic regions, as all locations fall under the same hospital using the same diagnostic and treatment protocols and all patients came from a small region.

Another limitation was the variability in intensity and kind of treatment within the TAU group. The number of sessions ranged from 3 to 37. Seven patients received intensive home treatment twice a week. The mean intensity between the two groups did not differ. In clinical practice, variability in intensity and treatment is very common, which is indicative that this group accurately reflects reality. And last, no follow-up was done after the 6 month study period. As autistic children require additional time to implement behavioral change, it is recommended to extend the follow-up period in future studies to 1 year.

### Generalizability

Patients were recruited from all referrals to outpatient clinics of Karakter. Patients could be referred by their family doctor, pediatricians or other medical specialists. Children of both sexes, aged between 3 and 8 years, with all comorbidity conditions (except unregulated ADHD), and who met the criteria for ASD could enroll. PRT is suitable for all age ranges. The techniques are equal, but the targets may differ.

## Conclusion

The present study suggests that PRT may lower severity of autism symptoms over TAU. Results should be replicated and extended in large scale randomized controlled trials.
